# Transcriptome Profiling Following Neuronal and Glial Expression of ALS-Linked SOD1 in *Drosophila*

**DOI:** 10.1534/g3.113.005850

**Published:** 2013-04-01

**Authors:** Emily L. Kumimoto, Taylor R. Fore, Bing Zhang

**Affiliations:** Department of Biology, University of Oklahoma, Norman, Oklahoma 73019

**Keywords:** *Drosophila*, ALS, SOD1, glia, motoneuron

## Abstract

Amyotrophic lateral sclerosis (ALS) generally is a late-onset neurodegenerative disease. Mutations in the Cu/Zn superoxide dismutase 1 (SOD1) gene account for approximately 20% of familial ALS and 2% of all ALS cases. Although a number of hypotheses have been proposed to explain mutant SOD1 toxicity, the molecular mechanisms of the disease remain unclear. SOD1-linked ALS is thought to function in a non–cell-autonomous manner such that motoneurons are critical for the onset, and glia contribute to progression of the disease. Recently, it has been shown in *Drosophila melanogaster* that expression of human SOD1 in a subset of neuronal cells causes synaptic transmission defects, modified motor function, and altered sensitivity to compounds that induce oxidative stress. Here we used the Gal4-UAS (Upstream Activation Sequence) system to further characterize flies expressing wild-type *Drosophila* SOD1 (dSOD1) and the mutant human SOD1^G85R^ (G85R) allele in motoneurons and glia. Cell-specific expression of both dSOD1 and G85R was found to influence lifespan, affect sensitivity to hydrogen peroxide, and alter lipid peroxidation levels. To better understand the genetic consequences of G85R expression in motoneurons and glia, we conducted microarray analysis of both young flies (5 days old) and old flies (45 days old) expressing G85R selectively in motoneurons or glia and concurrently in motoneurons and glia. Results from this microarray experiment identified candidate genes for further investigation and may help elucidate the individual and combined contributions of motoneurons and glia in ALS.

Amyotrophic lateral sclerosis (ALS), also known as Lou Gehrig's disease, involves the progressive degeneration of the cortical and spinal motoneurons (MN) that control voluntary skeletal muscle movement. Symptoms include muscle weakness, followed by paralysis as the disease progresses. Death results within 1–5 years after onset, usually due to respiratory failure (Boillee *et al.* 2006a). Most cases of ALS are sporadic, but approximately 10% are of familial origin, of which, approximately 20% have been linked to mutations in the Cu/Zn superoxide dismutase 1 (SOD1) gene (Boillee *et al.* 2006a; Rosen 1993).

SOD1 is ubiquitously expressed in all cells and functions to catalyze the dismutation of superoxide into oxygen and hydrogen peroxide. Over 150 mutations in the SOD1 gene have been linked to ALS. In mice, SOD1 loss-of-function does not result in neurodegeneration, whereas ubiquitous overexpression of mutant SOD1 leads to ALS-like symptoms, including those mutant SOD1 genes with a normal dismutase function (Bruijn *et al.* 1997; Gurney *et al.* 1994; Wong *et al.* 1995). This indicates that mutations result in a toxic gain of function, rather than loss of function, that leads to the selective degeneration of motoneurons. A number of hypotheses have been proposed to explain SOD1 toxicity, including oxidative stress, mitochondrial dysfunction, impaired axonal transport, glutamate excitotoxicity, protein aggregation, proteasome dysfunction, and others (Bruijn *et al.* 2004). However, the cellular and molecular mechanisms by which mutant SOD1 induces neurodegeneration is still not fully understood.

Although motoneurons are the cells that are primarily affected, it is believed that SOD1-linked ALS is a non–cell-autonomous disease and that glia contribute to the pathology. Disease symptoms fail to manifest in transgenic mice and rats expressing mutant SOD1 exclusively in either motoneurons or astrocytes (Gong *et al.* 2000; Lino *et al.* 2002). However, cell type selective excision of mutant SOD1 from motoneurons of transgenic mice delayed disease onset, while excision of mutant SOD1 from microglia slowed disease progression (Boillee *et al.* 2006b). Hence, these *in vivo* studies underscore the importance of glia as well as neuron-glia interaction in ALS development.

Flies and humans share highly conserved genes and similar cellular organization and functions of the nervous system, making *Drosophila* flies highly relevant to understanding human biology and disease. Human neurological diseases successfully modeled in flies include Alzheimer’s disease (Greeve *et al.* 2004; Iijima *et al.* 2004), Parkinson’s disease (Feany and Bender 2000; Greene *et al.* 2003; Yang *et al.* 2003), polyglutamine-related diseases (Jackson *et al.* 1998; Satterfield *et al.* 2002; Warrick *et al.* 1998), adrenoleukodystrophy (Min and Benzer 1999), fragile-X syndrome (Michel *et al.* 2004; Morales *et al.* 2002; Schenck *et al.* 2003; Tauber *et al.* 2011; Xu *et al.* 2004; Zhang *et al.* 2001), and autosomal dominant hereditary spastic paraplegia (Sherwood *et al.* 2004; Trotta *et al.* 2004). The use of flies to model these diseases has provided important insights into human neurological disorders, so flies may also make valuable contributions to understanding ALS.

Several attempts have been made at modeling SOD1-linked ALS in flies. Elia *et al.* (1999) expressed the G41S human SOD1 allele in motoneurons and found that it extended the lifespan of flies and enhanced resistance to paraquat (*N*,*N*′-dimethyl-4,4′-bipyridinium dichloride) (Elia *et al.* 1999). Mockett *et al.* (2003) ubiquitously expressed several mutant human SOD1 alleles (G37R, G41D, G93C, I113T, A4V) in SOD1-null mutant flies. In that case, however, only a recessive phenotype was observed that resulted in decreased lifespan and a sudden increase in oxidative stress in older flies. Using the human G85R and A4V alleles, Watson *et al.* (2008) demonstrated that expression of G85R in motoneurons impaired synaptic transmission, caused motor dysfunction, and resulted in accumulation of SOD1 aggregates. Recently, Islam *et al.* (2012) reported that cell-specific expression of G85R and A4V and wild-type human SOD1 altered motor function and influenced sensitivity to the neurotoxin β-*N*-methylamino l-alanine (BMAA). When these proteins were expressed under a Gal4 driver specific for glia, flies showed an age-dependent loss of motor function and increased sensitivity to BMAA. In contrast, when these proteins were selectively expressed in MN and simultaneously in MN and glia (MN+glia), motor activity was enhanced, and the flies had increased resistance to BMAA (Islam *et al.* 2012). These results indicate that the mutant allele used and the cell type in which it is expressed are important for investigating the effects of SOD1 in flies.

Here, we further investigated G85R expression in flies and report that cell-specific expression of wild-type *Drosophila* SOD1 and G85R influences lifespan, affects lipid peroxidation, and alters the sensitivity to hydrogen peroxide. To better understand the consequences of cell-specific expression of G85R, we conducted a microarray experiment to investigate global changes in gene expression in young and old flies expressing G85R in either MN or glia or in MN+glia. Results from this microarray experiment identified candidate genes for further investigation and may help elucidate the individual and combined contributions of motoneurons and glia in ALS.

## Materials and Methods

### *Drosophila* stocks and maintenance

We used the Gal4-UAS binary system (Brand and Perrimon 1993) to express wild-type *Drosophila* SOD1 (*dSOD1*) and mutant human SOD1 (*hSOD1*) in specific cell types. UAS-*dSOD1* (dSOD1) was used as a control , whereas the UAS-*hSOD1* gene carrying the G85R mutation (UAS-*hSOD1*^G85R^ [G85R]) is an ALS-linked hSOD1 mutant (Watson *et al.* 2008). As another control, the Gal4 drivers were crossed with a wild-type strain (Canton-S [CS]). D42-Gal4 (D42) (Yeh *et al.* 1995) and M1B-Gal4 (M1B) (Kretzschmar *et al.* 2005) were used to drive SOD1 expression in motoneurons and glia, respectively. D42-Gal4 and M1B-Gal4 (D42+M1B) were used together to express SOD1 in both motoneurons and glia simultaneously. For a green fluorescent protein (GFP) reporter, we used UAS-*mCD8GFP* (Lee and Luo 1999).

Adult male flies were collected within 24 hr after eclosion and placed into food vials in groups of 10. Stocks were reared on fly food (cornmeal-molasses-agar medium) under standard conditions (25°C, 70% humidity, and a 12-hr light/12-hr dark cycle) and transferred to fresh food vials every 5–7 days.

### Western blotting

To verify expression of SOD1 proteins, ten 1-day-old adult male flies were homogenized in 200 µl of 1% Triton-X 100. Samples were centrifuged at 13,000 × *g* for 5 min at 4°C. Sodium dodecyl sulfate (SDS) loading buffer was added to the supernatant and heated for 5 min at 100°C. Approximately 10 µg of protein was loaded onto a 15% SDS-polyacrylamide gel. Samples were separated by polyacrylamide gel electrophoresis and transferred to a nitrocellulose membrane. Western blots were probed as described by Watson *et al.* (2008). Specifically, we used the rabbit polyclonal hSOD1 antibody SC-11407 (catalog no. FL154,1:350 dilution; Santa Cruz Biotechnology, Santa Cruz, CA) to detect the dSOD1 protein and NCL-SOD1 mouse monoclonal antibody to hSOD1 (1:500 dilution; Novocastra Laboratories Ltd., Newcastle upon Tyne, UK) to detect G85R. Mouse monoclonal tubulin antibody was used as an internal reference (1:1000 dilution; product code, T6199; Sigma-Aldrich).

### Cell specificity of Gal4 drivers

To verify the cell-specific expression of the D42, M1B, and D42+M1B drivers, we crossed them with UAS-*mCD8GFP* as a reporter. F_1_ adults were collected and examined for GFP expression. The central nervous system (CNS) of adult males (<2 days after eclosion) was dissected in chilled 1× phosphate-buffered saline (PBS) and fixed in 4% formaldehyde, followed by a wash in 1× PBX (1× PBS containing 0.1% Triton-X). Primary antibodies were used as follows: anti-GFP (Invitrogen) at 1:1000 dilution; anti-Elav (monoclonal antibody (mAb): 9F8A9; Developmental Studies Hybridoma Bank [DHSB], University of Iowa; a pan-neuronal antibody) at 1:100 dilution; and anti-Repo (mAb: 8D12; DHSB; a glia-specific antibody) at 1:100 dilution. Secondary antibodies were labeled with Alexa Fluor 488 and 594 (Jackson ImmunoResearch) at 1:100 dilution. Fixed specimens were mounted in Vectashield (Vector Laboratories) and imaged using a Leica model TCS SP8 confocal microscope. Whole-brain images were taken using the tile scan feature and a plan-apochromat 25× (0.95 NA) lens at the following settings: 0.55-μm *z*-dimension steps, 1024 × 1024 pixels, 10% tile overlap, and 12-bit data acquisition. Maximum intensity *z* projections were generated from the merged tile scan image using FIJI software (Schindelin *et al.* 2012).

### Lifespan determination

Adult male flies were collected and maintained as described above. Flies were transferred to a new food vial and scored for survivorship every 2–3 days. Lifespan graphs were analyzed using GraphPad Prism 4 software, and *P* values were determined from a log-rank test.

### Hydrogen peroxide assay

Adult male flies were collected and maintained as described above. Flies were aged to 5 and 45 days (d) on fly food and then transferred to empty food vials containing a piece of filter paper saturated with 300 µl of 0.5% H_2_O_2_ in 3% sucrose. Flies were transferred to new vials containing fresh solution every 24 hr. Dead flies were counted every 12 hr. Survivorship graphs were analyzed in GraphPad Prism 4, and *P* values were determined from a log-rank test.

### Lipid peroxidation assay

Adult male flies were aged to 5 and 45 days old on fly food under standard conditions. Lipid peroxidation (LPO) levels were determined from three biological replicates, using the LPO-586 assay (catalog no. 21012; Bioxytech; Oxis Research). For each sample, 60 whole flies were homogenized in 300 µl of ice-cold 20 mM Tris, pH 7.4, with 5 mM butylated hydroxytoluene, and centrifuged for 10 min at 3000 × *g* at 4°C, and the supernatant was stored at −80°C. A 140-µl sample was added to 455 µl of reagent R1 (*N*-methyl-2-phenlindole in acetonitrile) diluted with ferric iron in methanol; and 105 µl of 12 N HCl was added to each sample and then incubated at 45° for 60 min. Samples were centrifuged for 10 min at 15,000 × *g* and 2× 300 µl of supernatant was transferred to a 96-well microplate. A Synergy HT plate reader (BioTek Instruments, Inc.) was used to measure absorbance at 586 nm, and samples were compared to 1,1,3,3-tetramethoxypropane standards. Results were analyzed using GraphPad Prism 4 software, and statistical significance was determined by one-way ANOVA, followed by a Bonferroni multiple comparison test.

### RNA preparation

Total RNA was isolated from three biological replicates for each genotype and each age for a total of 36 samples. Each replicate consisted of a pool of 40 adult male flies aged to 5 days and 45 days on cornmeal agar and then flash frozen in liquid nitrogen. Total RNA was isolated by homogenizing whole adult flies in Trizol (catalog no. 15596-026; Invitrogen), followed by a chloroform extraction (catalog no. CX1055; EMD Chemicals), isopropanol precipitation (catalog no. A414; Fisher Scientific), and an ethanol wash (catalog no. E200; Pharmco-AAPER). RNA pellets were resuspended in RNase-free water and purified using an RNeasy kit (catalog no. 74104; Qiagen) according to the manufacturer’s protocol. RNA concentration and purity were determined by analyzing the samples by spectrophotometry and with a bioanalyzer (model 2100 Bioanalyzer; Agilent Technologies).

### Microarray analysis

For each sample, 250 ng of total RNA was processed, labeled, and hybridized to Affymetrix GeneChip *Drosophila* Genome 2.0 arrays for a total of 36 arrays. The *Drosophila* Genome 2.0 array contains 18,800 probe sets, representing 18,500 transcripts (http://www.affymetrix.com). RNA labeling and GeneChip hybridizations were carried out by a service provider according to the manufacturer’s protocol (Nottingham *Arabidopsis* Stock Centre International Affymetrix Service, Loughborough, United Kingdom) (Craigon *et al.* 2004).

To analyze our microarrays, we used Robin, an open access graphic user interface that allows for R-based assessment and analysis of microarray data (Lohse *et al.* 2010). Cell intensity (CEL) files were imported into Robin version 1.1.5 software (http://mapman.gabipd.org), and, using the default settings, the data were normalized by robust multiarray averaging (RMA) (Irizarry *et al.* 2003), and linear models for microarray data (LIMMA) were selected as the analysis strategy (Lohse *et al.* 2010; Smyth 2004). Three chips consisting of independent biological replicates were analyzed for each genotype at each age with the exception of 5d M1B::G85R flies. Only 2 chips were used for analysis of 5d M1B::G85R flies because Robin detected RNA degradation in one of the replicates. Microarray data were deposited in the NCBI Gene Expression Omnibus (http://www.ncbi.nlm.nih.gov/geo/) under accession number GSE37148 (Barrett *et al.* 2011).

Changes in gene expression were first determined by comparing flies expressing G85R to their respective dSOD1 controls for each cell type at each age. To investigate the temporal effect of SOD1 expression, we performed a meta-analysis with Robin software. For the meta-analysis, 45d flies expressing G85R were first compared to their 5d G85R-expressing counterparts. Next, 45d control flies expressing dSOD1 were compared to their 5d dSOD1 counterparts. To eliminate the effects of normal aging, the transcriptional differences in expression in old G85R flies were then compared to the transcriptional differences of old dSOD1 flies. To correct for multiple comparisons, a Benjamini-Hochberg false discovery rate was applied to the data, and genes with a corrected *P* value of ≤0.05 were considered significant.

### Gene ontology and pathway analysis

Identification of enriched gene ontology (GO) terms was performed using genes with a Benjamini-Hochberg corrected *P* value of ≤0.05, using Database for Annotation, Visualization, and Integrated Discovery (DAVID) version 6.7 software (http://david.abcc.ncifcrf.gov) (Dennis *et al.* 2003; Huang *et al.* 2009). The background was set to the Drosophila_2 array, which corresponds to the Affymetrix GeneChip *Drosophila* Genome 2.0 array. The Functional Annotation Chart was used to identify enriched GO terms for biological processes, molecular functions, cellular components, and *Kyoto Encyclopedia of Genes and Genomes* (KEGG) pathways (Kanehisa and Goto 2000; Kanehisa *et al.* 2012). A Benjamini corrected Expression Analysis Systematic Explorer (EASE) score of *P* < 0.05 was considered statistically significant.

### Quantitative RT-PCR

To validate the microarray results, we performed quantitative RT-PCR (qPCR) experiments using an independent set of flies collected and maintained as described above. Samples consisted of three independent biological replicates containing pools of 40 whole, adult male flies. RNA was isolated as described above, and an on-column DNase treatment was performed during purification (catalog no. 79254; Qiagen). A 200-ng sample of RNA was reverse transcribed to cDNA using Superscript III first-strand synthesis SuperMix for qRT-PCR according to the manufacturer’s protocol (catalog no. 11752-050; Invitrogen). qPCR was performed with a model 7500 real-time PCR system (Applied Biosystems) using Maxima SYBR Green/ROX qPCR Master Mix (catalog no. K0222; Fermentas). Cycling conditions were 2 min at 50°C, then 10 min at 95°C, followed by 40 cycles of 15 sec at 95°C, and 1 min at 60C°. Ribosomal protein L32 (rp49) was used as the reference gene to control for error between samples. Primer3 was used for primer design (Rozen and Skaletsky 2000) (Supporting Information, Table S1). Relative changes in gene expression were calculated using the 2^−ΔΔCT^ method (Livak and Schmittgen 2001). Gene expression changes in G85R flies are relative to dSOD1 expression under the same cell-specific Gal4 drivers. Statistical significance was determined by a *t*-test, and *P* < 0.05 was considered significant.

## RESULTS

### Expression of SOD1 is cell-specific

**Figure 1:**
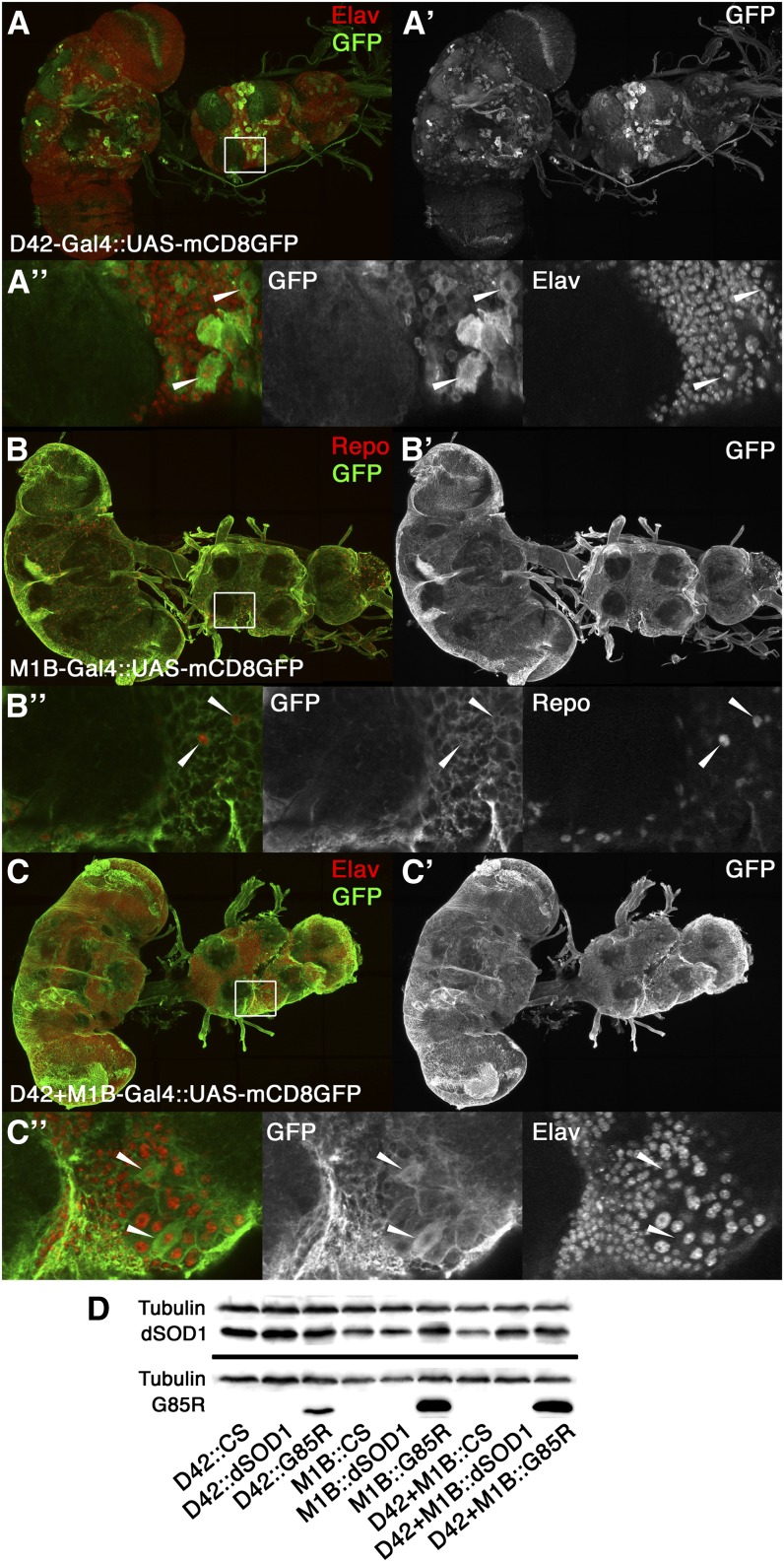
Cell specificity of Gal4 drivers and transgene expression of dSOD1 and G85R. Cell-specific expression was confirmed by crossing Gal4 drivers with a green fluorescent protein (GFP) reporter (UAS-*mCD8GFP*). Protein expression was confirmed by Western blot analysis. (A, B, C) Maximum intensity projections of motoneurons (D42) and glia-specific (M1B) and combined D42+M1B Gal4 drivers expressing membrane-bound GFP (A′, B′, and C′). Neuronal and glia nuclei (in red) are anti-Elav and anti-Repo markers, respectively. (A,” B,” C”) Selected z slices from the T1 segment of the ventral nerve cord (VNC). (A”) Arrowheads show the cell body of motoneurons. (B”) GFP is extensively expressed in all glial processes, with select glia nuclei. (C”) In the combined driver, motor neuron cell bodies are morphologically distinct among the glia processes. (D) Western blot analysis showing expression of the G85R transgene under the D42, M1B, and D42+M1B Gal4 drivers. Extracts from 1-day-old flies were probed with antibodies that detect dSOD1 (FL154), hSOD1 (NCL-SOD1), and tubulin (serving as a loading control).

Although the Gal4 drivers used in our experiments are well-established by other studies (Kretzschmar *et al.* 2005; Parkes *et al.* 1998; Yeh *et al.* 1995), we sought to confirm their cell expression specificity by crossing the D42, M1B, and D42+M1B Gal4 drivers with a UAS-*mCD8GFP* reporter (Figure 1, A-C). Our data confirmed that D42 is specific to motoneurons in both the ventral nerve cord and brain, whose nuclei were positive for the neuronal marker Elav (Robinow and White 1991). M1B expresses specifically in glia with elaborate processes, whose nuclei are positive for the glial marker Repo (Halter *et al.* 1995; Xiong *et al.* 1994). We also used Western blot analysis to confirm the presence of dSOD1 and G85R proteins in 1d flies when crossed with the D42, M1B, and D42+M1B Gal4 drivers (Figure 1D). G85R expression was confirmed using an antibody that detects only hSOD1 (NCL-SOD), and *Drosophila* SOD1 expression was detected with an antibody for dSOD1 (code FL154) (Watson *et al.* 2008).

### SOD1 expression affects fly lifespan in a cell-specific manner

**Figure 2:**
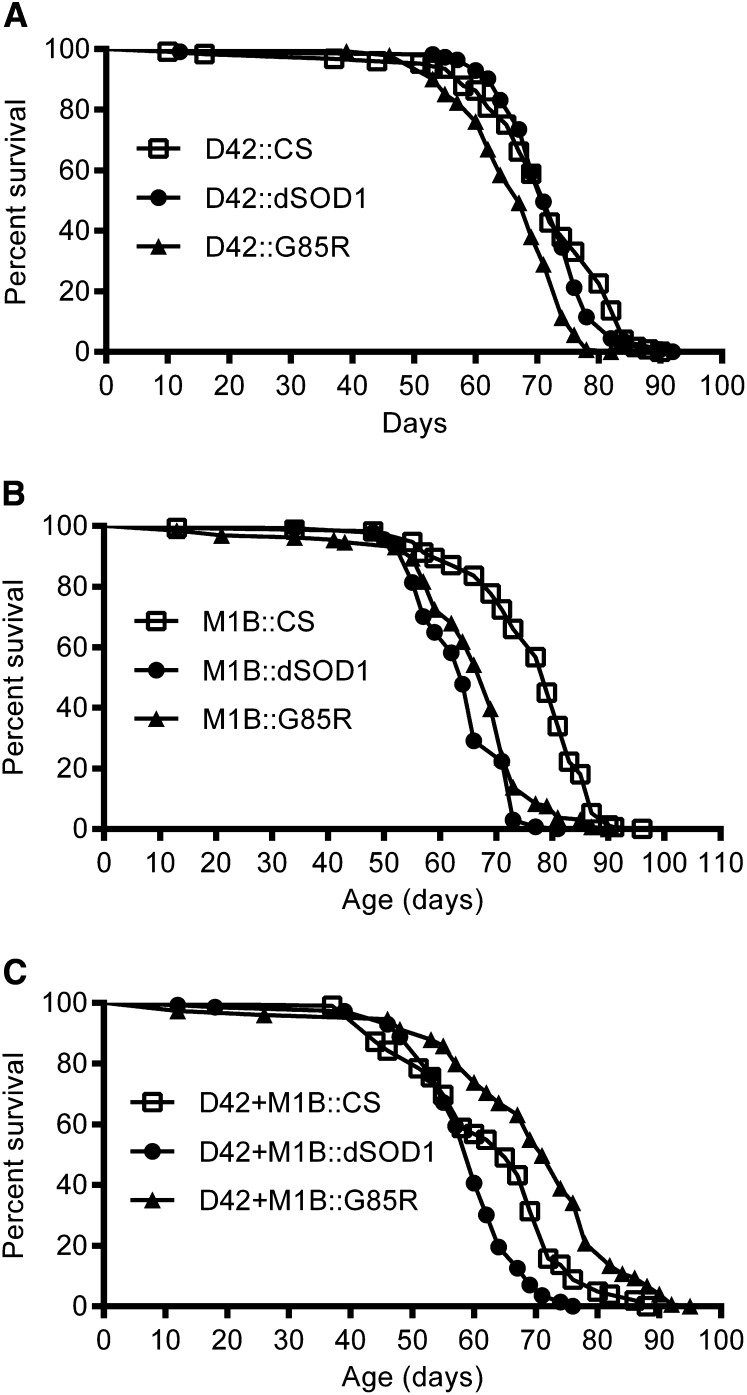
Lifespan of flies with cell-specific Gal4 drivers crossed with wild-type (CS), dSOD1, and G85R flies. (A) Lifespan of flies with the D42 motoneuron Gal4 driver (D42::CS, n = 125; D42::dSOD1, n = 113; and D42::G85R, n = 142). D42::dSOD1 flies had a lifespan similar to that of D42::CS flies. D42::G85R flies had a reduced lifespan compared to that of D42::dSOD1 (*P* < 0.0001) and D42::CS flies (*P* < 0.0001). (B) Lifespan of flies with the M1B glia Gal4 driver (M1B::CS, n = 172; M1B::dSOD1, n = 165; and M1B::G85R, n = 138). M1B::dSOD1 flies had a reduced lifespan compared to that of M1B::CS flies (*P* < 0.0001). M1B::G85R flies had a reduced lifespan compared to that of M1B::CS flies (*P* < 0.0001) and a slightly increased lifespan compared to that of M1B::dSOD1 flies (*P* < 0.01). (C) Lifespan of flies with D42+M1B Gal4 driver (D42+M1B::CS, n = 102; D42+M1B::dSOD1, n = 143; and D42+M1B::G85R, n = 148). D42+M1B::dSOD1 flies had a reduced lifespan compared to that of D42+M1B::CS flies (*P* < 0.0001). D42+M1B::G85R flies had an increased lifespan compared to those of both D42+M1B::CS flies (*P* < 0.0001) and D42+M1B::dSOD1 flies (*P* < 0.0001).

Transgenic expression of neurodegenerative disease proteins in flies typically shortens the lifespan of flies (Finelli *et al.* 2004; Lee *et al.* 2004; Miguel *et al.* 2011; Pandey and Nichols 2011; Yang *et al.* 2006). To examine the effect of cell-specific expression of G85R on fly lifespan, we used D42, M1B, and D42+M1B Gal4 drivers to express dSOD1 (control) or G85R (experimental) in MN, glia, and MN+glia, respectively. As an additional control, we also crossed the Gal4 drivers with the wild-type CS strain. Expression of G85R in MN (D42::G85R) slightly decreased the lifespan of male flies compared to those of D42::dSOD1 and D42::CS controls (Figure 2A). The 50% survival time (S50) was 67 days for D42::G85R flies, 71 days for D42::dSOD1 flies, and 72 days for D42::CS flies. Expression of G85R in glia (M1B::G85R) had a lifespan similar to M1B::dSOD1 controls (S50 = 69 *vs*. S50 = 64, respectively). However, both M1B::G85R and M1B::dSOD1 treatments decreased the lifespan of flies compared to that with M1B::CS (S50 = 79) (Figure 2B). Surprisingly, simultaneous expression of G85R in MN+glia (D42+M1B::G85R) increased lifespan of flies (S50 = 71) compared to D42+M1B::dSOD1 (S50 = 60) and D42+M1B::CS (S50 = 65) controls. These results indicate that overexpression of G85R in motoneurons or glia, and not in both, decreased the lifespan of flies. Similar to M1B::dSOD1, D42+M1B::dSOD1 decreased lifespan compared to their respective CS controls (Figure 2C), suggesting that overexpression of the wild-type dSOD1 in glia may also affect flies.

### Older flies expressing mutant human SOD1 exhibit an increased sensitivity to hydrogen peroxide

**Figure 3:**
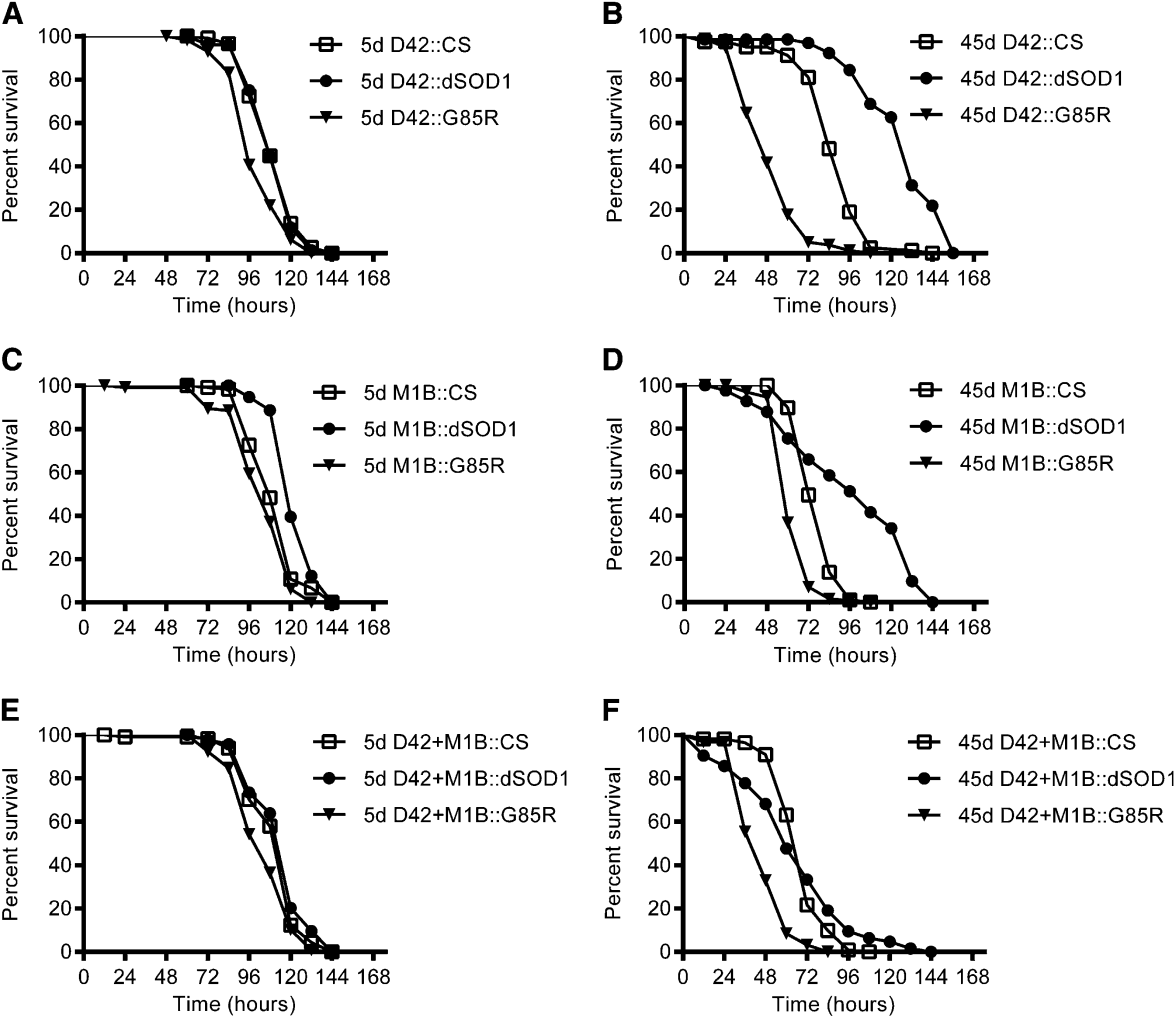
Older flies expressing G85R were more sensitive to low levels of hydrogen peroxide. All flies were fed on 0.5% H_2_O_2_ in 3% sucrose. (A) Survivorship graphs of 5-day-old flies with motoneuron D42 Gal4 driver (D42::CS, n = 116; D42::dSOD1, n = 76; and D42::G85R, n = 113). D42::G85R flies were slightly more sensitive to H_2_O_2_ than D42::CS (*P* < 0.0001) and D42::dSOD1 (*P* < 0.0001) flies. (B) Survivorship graphs of 45-day-old flies with D42 Gal4 driver (D42::CS, n = 79; D42::dSOD1, n = 65; and D42::G85R, n = 79). D42::dSOD1 flies had increased resistance to H_2_O_2_ compared to D42::CS flies (*P* < 0.0001). D42::G85R flies had increased sensitivity to H_2_O_2_ compared to D42::CS (*P* < 0.0001) and D42::dSOD1 (*P* < 0.0001) flies. (C) Survivorship graphs of 5-day-old flies with glia M1B Gal4 driver (M1B::CS, n = 120; M1B::dSOD1, n = 114; M1B::G85R). M1B::dSOD1 flies were slightly more resistant to H_2_O_2_ than M1B::CS (*P* < 0.0001) and M1B::G85R (*P* < 0.0001) flies. (D) Survivorship graphs of 45-day-old flies with M1B driver (M1B::CS, n = 172; M1B::dSOD1, n = 41; M1B::G85R, n = 87). M1B::dSOD1 flies had increased resistance to H_2_O_2_ compared to M1B::CS flies (*P* < 0.01) flies. M1B::G85R flies had increased sensitivity to H_2_O_2_ compared to M1B::CS (*P* < 0.0001) and M1B::dSOD1 (*P* < 0.0001) flies. (E) Survivorship graphs of 5-day-old flies with D42+M1B Gal4 driver (D42+M1B::CS, n = 114, D42+M1B::dSOD1, n = 94; D42+M1B::G85R, n = 118). D42+M1B::G85R flies were slightly more sensitive to H_2_O_2_ than D42+M1B::CS (*P* < 0.01) and D42+M1B::dSOD1 (*P* < 0.0001) flies. (F) Survivorship graphs of 45-day-old flies with D42+M1B Gal4 driver (D42+M1B::CS, n = 111; D42+M1B::dSOD1, n = 63; D42+M1B::G85R, n = 94). D42+M1B::dSOD1 flies had a sensitivity similar to that of H_2_O_2_ as D42+M1B::CS flies. D42+M1B::G85R flies were more sensitive to H_2_O_2_ than D42+M1B::CS (*P* < 0.0001) and D42+M1B::dSOD1 (*P* < 0.0001) flies.

Environmental factors are thought to contribute to ALS and other neurodegenerative diseases (Mitchell 2000). To test the effect of environmental oxidative stress, we fed 5d and 45d flies 0.5% H_2_O_2_ in 3% sucrose (Figure 3). At 5d, D42::G85R flies (S50 = 96 hr) were slightly more sensitive to H_2_O_2_ than D42::CS (S50 = 108 hr) or D42::dSOD1 (S50 = 108 hr). Under the glia driver, 5d M1B::dSOD1 flies (S50 = 120 hr) were slightly more resistant to H_2_O_2_ than M1B::CS (S50 = 108 hr) and M1B::G85R (S50 = 108 hr); 5d D42+M1B::G85R flies (S50 = 108 hr) were also slightly more sensitive to H_2_O_2_ than D42+M1B::CS (S50 = 120 hr) and D42+M1B::dSOD1 (S50 = 120 hr). All 45d G85R-expressing flies had an increased sensitivity compared to both their respective dSOD1 and CS controls as measured by the effect of H_2_O_2_ on the lifespan of flies. The S50 was 48 hr for D42::G85R, 84 hr for D42::CS, and 132 hr for D42:dSOD1. With glial expression, M1B::G85R also had the shortest lifespan (S50 = 60 hr), followed by M1B::CS (S50 = 72 hr) and M1B::dSOD1 (S50 = 108 hr). Under the control of MN+glia Gal4 drivers, the lifespan of G85R flies was also shorter (S50 = 48 hr) than that of D42+M1B::dSOD1 (S50 = 60 hr) and D42+M1B::CS (S50 = 72 hr). Interestingly, D42::dSOD1 and M1B::dSOD1 increased resistance to H_2_O_2_ compared to their respective CS controls, but D42+M1B::dSOD1 flies did not. Furthermore, expression of dSOD1 in glia or MN+glia also had a complex effect on lifespan, with notable early deaths but prolonged survival in later hours. Overall, when exposed to H_2_O_2_, G85R expression significantly shortened the lifespan of flies in MN, glia, or both compared to either the CS control or its genetically closer background control (dSOD1).

### Older flies with G85R expression in MN+glia had increased levels of lipid peroxidation

**Figure 4:**
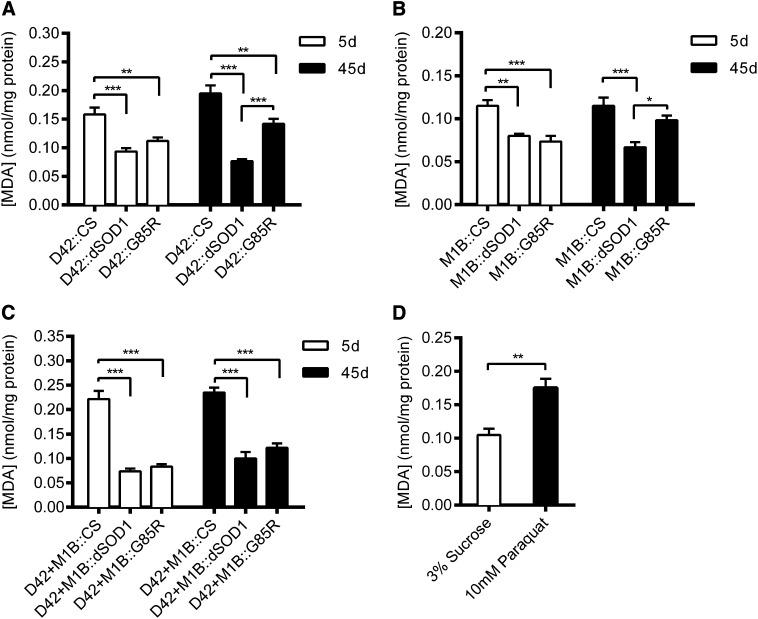
Lipid peroxidation levels of 5- and 45-day-old flies with cell-specific Gal4 drivers crossed with wild-type (CS), dSOD1, and G85R flies, as measured by malondialdehyde (MDA) concentration (nmol/mg). (A) MDA for flies with the D42 motoneuron Gal4 driver. (B) [MDA] for flies with the M1B glia Gal4 driver. (C) [MDA] for flies with the D42+M1B motoneuron and glial Gal4 drivers. (D) [MDA] for 5-day-old wild-type (CS) flies treated with 10 mM paraquat in 3% sucrose solution or the sucrose solution. *P < 0.05; **P < 0.001; ***P < 0.0001.

Oxidative stress is believed to be an important mechanism in the pathology of ALS (Carri *et al.* 2003). Lipids are susceptible to oxidative degradation, and the end products of their degradation can be quantified as a measure of oxidative stress. Here, we measured malondialdehyde (MDA) concentration to test flies with cell-specific expression of G85R for indications of oxidative stress (Figure 4, A–C). Cell-specific Gal4 drivers crossed with wild-type (CS) flies had higher MDA levels than those with either dSOD1 or G85R. Relative to the Gal4 driver::CS, Gal4::dSOD1 is a closer genetic control for Gal4::G85R. Hence, we compared dSOD1 and G85R for their effects on MDA levels. There were no statistical differences in MDA levels between any 5d flies expressing G85R and their dSOD1 controls. At 45d, however, D42::G85R and M1B::G85R had significantly increased MDA levels compared to their respective dSOD1 controls. D42+M1B::G85R flies, however, were not significantly different from D42+M1B::dSOD1 controls. To ascertain that we could detect changes in lipid peroxidation, we fed 5d wild-type flies with paraquat (10 mM in 3% sucrose solution) and detected a significant increase in MDA levels compared to CS flies fed a 3% sucrose control solution (Figure 4D).

### Overview of gene expression profile

To better understand the genetic consequences of G85R expression in neuronal cells, we conducted a microarray experiment using flies with G85R expression in MN, glia, or in MN+glia. RNA was isolated from whole, adult flies aged 5d and 45d. We selected these ages because previous studies revealed that synaptic transmission along the giant fiber pathway was disrupted in older G85R flies (Watson *et al.* 2008). The genetic cross schemes for the microarray are outlined in Table S2.

We analyzed our microarray data in several ways. First, we determined differences in gene expression by comparing flies expressing G85R to their respective dSOD1 controls for each age. We then examined those genes with the greatest transcriptional changes (≥2-fold) to identify potential hits for future examination. Next, we performed enrichment analysis to identify biological themes that might be overrepresented in flies expressing G85R. Symptoms of ALS do not manifest until later in life and may be the result of cumulative damage that occurs over many years. Therefore, we performed a meta-analysis where we examined the effect of G85R expression in 45d flies and compared it to 5d flies and subtracted the effect of aging in the dSOD1 controls. Finally, we compared our *Drosophila* microarray results to several SOD1 mouse and ALS human patient microarrays to identify genes that may be commonly misregulated.

### Genes with the greatest changes in expression

**Figure 5:**
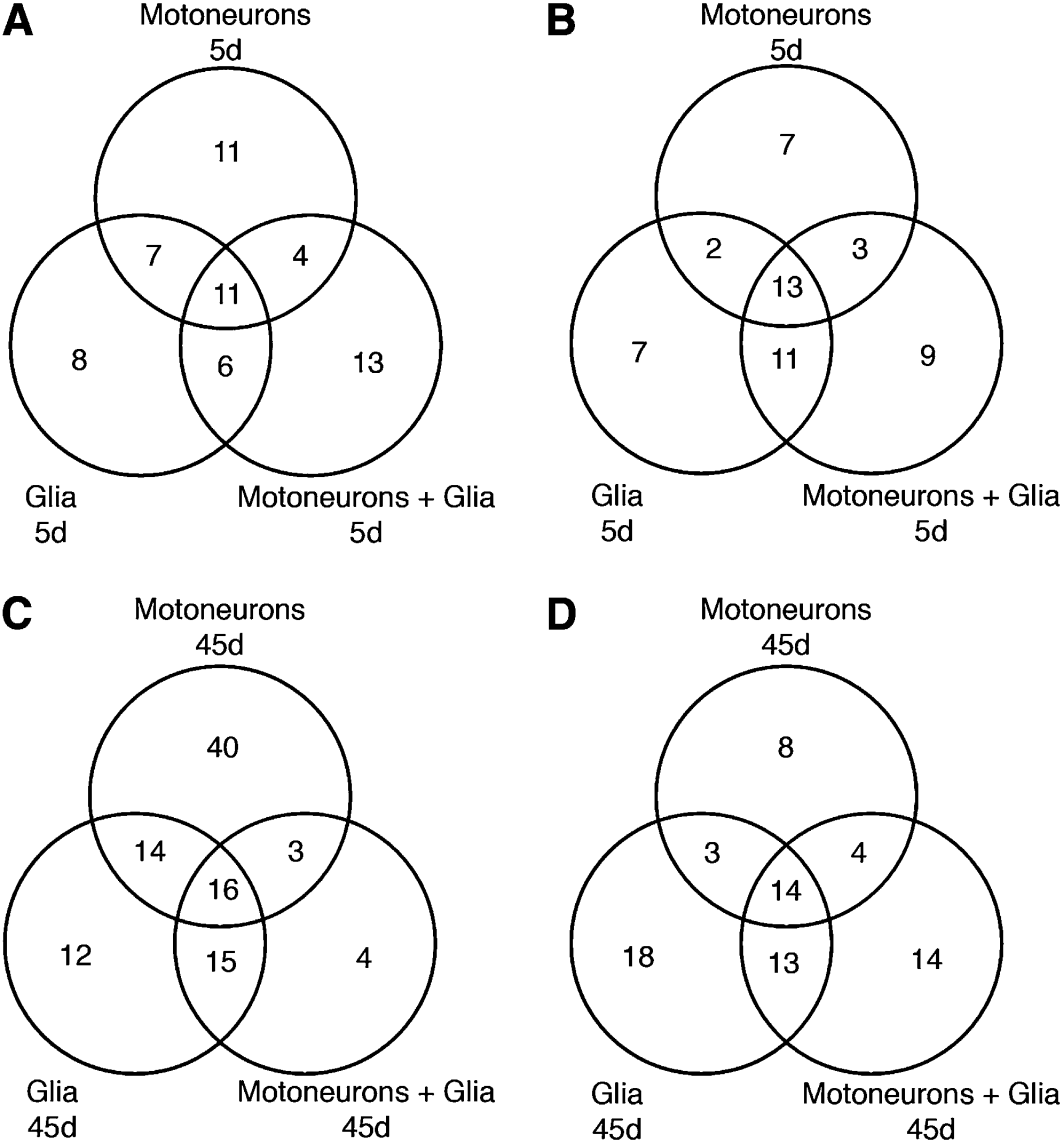
Venn diagrams showing overlap in differentially expressed genes with at least a two-fold change when G85R was expressed in motoneurons, glia, or together in both cell types. (A) Up-regulated genes in 5-day-old flies. (B) Down-regulated genes in 5-day-old flies. (C) Up-regulated genes in 45-day-old flies. (D) Down-regulated genes in 45-day-old flies.

To identify target genes for further investigation, we compiled a list of those genes with the highest changes in expression levels (≥2-fold) (File S1). When G85R was expressed only in MN, 33 transcripts with at least twofold change were up-regulated, and 25 transcripts were down-regulated in 5d flies. As these flies aged to 45d, 73 transcripts were up-regulated, and 29 transcripts were down-regulated. When G85R was expressed only in glia, 32 transcripts were up-regulated, and 33 transcripts were down-regulated in 5d flies, whereas 57 transcripts were up-regulated and 48 transcripts were down-regulated in 45d flies. When G85R was expressed simultaneously in MN+glia, 34 transcripts were up-regulated, and 36 transcripts were down-regulated in 5d flies, while 38 transcripts were up-regulated and 45 transcripts were down-regulated in 45d flies. As illustrated in a Venn diagram, overlap in genes with altered expression was observed (Figure 5).

Broadly, the list of genes whose expression was affected by cell-specific expression of G85R includes those involved with oxidative stress, mitochondria, lipid metabolism, and, of particular interest, a number of neurodevelopmental and signaling genes. Transcriptional changes in several of these genes are verified by qPCR using an independent set of flies (File S2). Genes that were confirmed from the microarray results included the following (↑ indicates up-regulation, and ↓ indicates down-regulation): CG13551 (↓5d MN+glia) is involved with the defense response (Feng *et al.* 2009) and is also predicted to be a mitochondrial ATPase inhibitor (Tweedie *et al.* 2009). CG31742 (↓5d, ↓45d MN, glia, MN+glia) is a proteasome beta subunit that is a component of the 20S catalytic core of the 26S proteasome and has a role in the response to DNA damage (Ravi *et al.* 2009). CG33296 (↑5d, ↑45d MN, glia, MN+glia) is predicted to be a neurotransmitter transporter (Thimgan *et al.* 2006; Tweedie *et al.* 2009). Protein tyrosine phosphatase 99A (Ptp99A; ↓5d MN+glia) is part of the phosphotyrosine signaling pathway involved with axon guidance (Desai *et al.* 1996). Rhomboid (Rho; ↓45d glia, MN+glia) is a serine protease that regulates epidermal growth factor signaling by cleaving the growth factor Spitz (Urban *et al.* 2001) and plays a role in the development of the nervous system (Bier *et al.* 1990; Salzberg *et al.* 1994; Sepp and Auld 2003). SCAP (↓45d MN, ↓5d, ↓45d glia, MN+glia) is a regulatory protein that regulates cleavage of the sterol regulatory element-binding protein (SREBP) to maintain fatty acid homeostasis (Seegmiller *et al.* 2002). Slamdance (Sda; ↓45d glia, MN+glia) is the *Drosophila* homolog of the human aminopeptidase N gene and has a role in nervous system excitability (Marley and Baines 2011; Zhang *et al.* 2002). In flies, *sda* mutants are prone to paralysis and seizures after exposure to mechanical and electric shock (Marley and Baines 2011; Pavlidis and Tanouye 1995; Zhang *et al.* 2002). Tumbleweed (Tum; ↑5d, ↑45d MN, glia, MN+glia) is a Rho family GTPase-activating protein (Goldstein *et al.* 2005) and is involved in several processes such as cytokinesis, where it regulates microtubule bundling (Somers and Saint 2003), dendrite morphogenesis (Gao *et al.* 1999), neuroblast proliferation, and axon development (Goldstein *et al.* 2005) and has a role in Wnt regulation (Jones and Bejsovec 2005; Jones *et al.* 2010). Wrapper (↑45d glia, MN+glia) is a member of the immunoglobulin superfamily that is expressed on the surface of midline glia and plays a role in axonal ensheathment (Noordermeer *et al.* 1998; Wheeler *et al.* 2009).

### Gene ontology enrichment analysis

We next performed enrichment analysis of the microarray data using DAVID software, where up- and down-regulated genes were analyzed separately (File S3). Because mRNA was extracted from whole flies, the signal of many potentially interesting transcripts may have been diluted and consequently missed by assigning arbitrary fold change cutoff values when we evaluated the data. Therefore, changes in expression of transcripts with statistical significance levels with a Benjamini-Hochberg correction value of *P* ≤ 0.05, without regard to fold change, were used to identify overrepresented terms. GO terms with a Benjamini corrected EASE score of *P* < 0.05 were considered statistically significant. When G85R was expressed in MN, 879 genes were up-regulated in 5d flies, and 724 genes were down-regulated in 5d flies. In 45d flies with G85R in MN, 1011 genes were up-regulated, and 928 genes were down-regulated. When G85R was expressed in glia, 535 genes were up-regulated in 5d flies, and 571 genes were down-regulated in 5d flies. In 45d flies with G85R in glia, 952 genes were up-regulated, and 985 genes were down-regulated. When G85R was expressed in MN+glia, 590 genes were up-regulated in 5d flies, and 701 genes were down-regulated in 5d flies. In 45d flies with G85R in MN+glia, 577 genes were up-regulated, and 776 genes were down-regulated.

#### GO terms unique to MN:

Several GO terms were uniquely enriched when G85R was expressed in motoneurons. In the down-regulated set of genes for 5d flies, the GO term for mitochondrion in the cellular component category was enriched. Mitochondria are the primary site of ATP production, maintain calcium homeostasis, and have a role in apoptosis. Mitochondrial dysfunction has been implicated in several neurodegenerative diseases and is thought to play a key role in ALS. Dilated and vacuolated mitochondria have been observed in ALS patients and transgenic mice (Afifi *et al.* 1966; Dal Canto and Gurney 1994; Higgins *et al.* 2002; Higgins *et al.* 2003; Hirano *et al.* 1984a; Hirano *et al.* 1984b; Kong and Xu 1998; Wong *et al.* 1995), and mitochondrial abnormalities have been reported in the early stages of the disease (Higgins *et al.* 2003; Kong and Xu 1998; Wong *et al.* 1995). Mutant SOD1 has also been shown to colocalize to the mitochondria, where it may induce stress and interfere with proper functioning of the organelle (Deng *et al.* 2006; Higgins *et al.* 2002; Liu *et al.* 2004; Vijayvergiya *et al.* 2005).

In 45d flies expressing G85R in MN, the biological processes of NADP metabolism and nicotinamide metabolism were enriched among the down-regulated set of genes. Nicotinamide is a precursor to nicotinamide adenosine dinucleotide (NAD) and nicotinamide adenine dinucleotide phosphate (NADP), coenzymes with important roles in redox reactions. NAD has a role in energy metabolism, whereas NADP plays a role in cholesterol and lipid synthesis and fatty acid chain elongation and in redox reactions that protect against reactive oxygen species (ROS).

#### GO terms unique to glia:

In the up-regulated set of genes in 45d flies expressing G85R in glia, GO terms for the biological processes of muscle system process and muscle contraction were enriched, and GO terms for the cellular components contractile fiber and contractile fiber parts were enriched. These GO terms refer to the generation of force in a muscle and the components of contractile muscle fibers, such as actin, myosin, and associated proteins. There is evidence that muscle degeneration occurs early in ALS where it may precede motoneuron degeneration (Brooks *et al.* 2004; Marcuzzo *et al.* 2011) and may be an indication of degeneration occurring at the neuromuscular junction (Chiu *et al.* 1995; Frey *et al.* 2000; Kennel *et al.* 1996). Recently, Islam *et al.*(2012) reported that flies expressing mutant human SOD1 in glia showed an age-dependent loss of motor function in a negative geotaxis assay. In contrast, mutant SOD1 expression in MN and coexpression in MN+glia enhanced climbing ability in flies.

In the down-regulated genes for 45d flies expressing G85R in glia, GO terms for the molecular function of glutathione transferase activity and the KEGG pathway for glutathione metabolism were enriched. Glutathione transferases are a family of enzymes that are involved with the detoxification process. Glutathione has many important functions in cells and is a major endogenous antioxidant that directly neutralizes ROS.

#### GO terms unique to MN+glia:

Enriched GO terms unique to flies simultaneously expressing G85R in MN+glia includes the molecular function of neuropeptide hormone activity from the list of up-regulated genes in 45d flies. Neuropeptides are signaling molecules in neurons that regulate physiology and behavior.

In the down-regulated list of genes for 45d with MN+glia expression of G85R, septate junction assembly was enriched. Septate junctions are the invertebrate equivalent of vertebrate tight junctions where they provide structural support to cells and act as a permeability barrier. Dysfunction of the blood-brain barrier, which is maintained by tight junctions, is thought to contribute to neurodegenerative diseases, including ALS (Rodrigues *et al.* 2012).

#### GO terms shared by MN+glia:

While some GO terms were uniquely enriched when G85R was expressed under a certain cell-specific driver, others were commonly enriched among several or all cell type expression patterns of G85R. The pentose-phosphate KEGG pathway was enriched in the down-regulated set of genes in 45d flies expressing G85R in MN and in glia. The pentose phosphate pathway generates pentoses and is the main source of NADPH, which is used as a reducing equivalent for biosynthetic reactions and oxidation-reduction reactions that protect against ROS.

#### GO terms shared by MN and MN+glia:

GO terms for the biological process of immune and defense response were enriched in the up-regulated set of genes in 45d flies with G85R expression in MN and MN+glia. Neuroinflammation is characteristic of certain neurodegenerative diseases, including ALS. (Papadimitriou *et al.* 2010). Within the central nervous system, activated microglia and astrocytes are commonly observed in ALS (Alexianu *et al.* 2001; Hall *et al.* 1998; Mcgeer and Mcgeer 2002). In the SOD1 mouse model, activation of the innate and humoral immune systems has also been demonstrated in the peripheral nervous system (Chiu *et al.* 2009).

The KEGG pathway for folate biosynthesis was enriched in the up-regulated set of genes in 45d flies expressing G85R in MN and in MN+glia. Folate has an important role in maintaining low levels of homocysteine, which is cytotoxic at high levels. Homocysteine is produced from the demethylation of methionine and folate reduces homocysteine levels by promoting its remethylation. Elevated homocysteine levels are a risk factor for several neurodegenerative disorders, including ALS (Diaz-Arrastia 2000; Zoccolella *et al.* 2008) and may serve as a potential biomarker for ALS. Additionally, decreased folic acid has been observed in ALS patients as well as the SOD1 mouse model (Zhang *et al.* 2010; Zoccolella *et al.* 2008).

#### GO terms shared by MN, glia, and MN+glia:

Several enriched GO terms were common to flies expressing G85R under the MN, glia, and MN+glia Gal4 drivers. With the exception of 5d MN and 45d MN+glia gene lists, the GO term for the biological process of proteolysis was enriched in all up-regulated gene lists. Abnormal protein aggregates are a common feature of ALS and other neurodegenerative diseases. SOD1 protein aggregates have been observed to accumulate in the tissues of the central nervous system of FALS patients and mutant SOD1 transgenic mice and flies (Bruijn *et al.* 1997; Johnston *et al.* 2000; Shibata *et al.* 1996; Watanabe *et al.* 2001; Watson *et al.* 2008). Misregulation of the ubiquitin-proteasome pathway is thought to be involved in the formation of mutant SOD1 aggregates and, as demonstrated by Puttaparthi *et al.* (2003), even a 30% reduction in proteasomal activity results in mutant SOD1 aggregate formation.

The GO term for the biological process of oxidation-reduction was enriched in all down-regulated gene lists except 5d flies with MN expression of G85R. Oxidation-reduction (redox) reactions are chemical reactions that involve the gain or loss of electrons by an atom, ion, or molecule. Redox reactions are the basis for many biochemical pathways and are also important for understanding an organism’s ability to cope with ROS. Imbalances in redox state can result in excessive ROS and lead to oxidative stress (Kohen and Nyska 2002).

### Meta analysis

ALS is a neurodegenerative disease that usually occurs later in life. To examine the temporal effects of cell-specific expression of G85R in flies, we performed a meta-analysis of the data (File S4). First, we compared 45d flies expressing G85R to their 5d G85R counterparts. Next, we compared 45d control flies expressing dSOD1 to their 5d dSOD1 counterparts. To eliminate the effects of normal aging, we then compared the transcriptional differences in expression in old G85R flies to the transcriptional differences of old dSOD1 flies. In old flies with MN expression of G85R, GO terms for defense response and mitochondria were enriched in the up-regulated set of genes while oxidation reduction was enriched in the down-regulated set of genes. In old flies with glial expression of G85R, no GO terms were significantly enriched in the up-regulated set of genes. GO terms enriched in the down-regulated set of genes include oxidation reduction, fatty acid metabolism, and glutathione metabolism. When G85R was expressed simultaneously in MN+glia, no GO terms were significantly enriched in the up-regulated set of genes for old flies. GO terms enriched in the down-regulated set of genes include septate junctions, glial cell development, and proteolysis.

### Comparison of array results obtained from *Drosophila* and other SOD1 animal models and ALS human patients

Compared to several other ALS microarrays (Brockington *et al.* 2010; Chen *et al.* 2010; Cox *et al.* 2010; D’arrigo *et al.* 2010; Ferraiuolo *et al.* 2007; Ferraiuolo *et al.* 2011; Gonzalez De Aguilar *et al.* 2008; Jiang *et al.* 2005; Kirby *et al.* 2005; Kudo *et al.* 2010; Vargas *et al.* 2008; Yoshihara *et al.* 2002), we identified five genes from our ≥2-fold change list that have orthologs in SOD1 transgenic mice and human patients with sporadic ALS (Table 1). Sepia is the fly ortholog of mouse glutathione S-transferase omega 1 (O’brien *et al.* 2005) and was up-regulated in 45d flies with G85R in glia and MN+glia but was down-regulated in mouse motoneurons (Kirby *et al.* 2005). Pka-C1 is the fly ortholog of mouse protein kinase, cAMP-dependent, catalytic, alpha (O’brien *et al.* 2005) and was down-regulated in G85R flies, especially in those with glial expression, and was also down-regulated in mouse astrocytes. *sfl* is the ortholog of human *N*-deacetylase/*N*-sulfotransferase (heparan glucosaminyl) 2 (O’brien *et al.* 2005) and was down-regulated in G85R flies but up-regulated in the motoneurons of human ALS patients (Jiang *et al.* 2005). *CG31742* shares sequence similarity to human proteasome (prosome, macropain) subunit, beta type 5 and was down-regulated in G85R flies but up-regulated in the ventral horn of ALS human patients (Jiang *et al.* 2005). *sda* is the ortholog of human alanyl (membrane) aminopeptidase (Zhang *et al.* 2002) and was down-regulated in 45d flies which expressed G85R in MN and it was also down-regulated in the ventral horn of ALS human patients (Jiang *et al.* 2005).

**Table 1:** Mammalian Orthologs of *Drosophila* Genes Affected by Mutant SOD1 Expression

Probe Set ID	Fly Gene	5-Day-Old Flies Fold Change	45-Day-Old Flies Fold Change	Cell Type Expression of G85R	Ortholog	Organism	Fold Change	Tissue for Microarray (study ref.)
1637801_at	Sepia	1.12*^a^*	−1.01*^a^*	MN	Glutathione S-transferase omega 1	mouse	−3.02	Motoneurons (Kirby *et al.* 2005)
		1.22*^a^*	2.77	Glia				
		1.05*^a^*	2.78	MN+glia				
1631055_at	cAMP-dependent protein kinase 1	−1.20*^a^*	−1.26*^a^*	MN	Protein kinase, cAMP- dependent, catalytic, alpha (Prkaca)	mouse	−2.11	Astrocytes (Ferraiuolo *et al.* 2011)
		−1.93	−2.10	Glia				
		−1.23*^a^*	−1.45	MN + glia				
1631534_at	Sulfateless	−1.87	−1.71	MN	*N*-deacetylase/*N*-sulfotransferase (heparan glucosaminyl) 2	human	1.52	Motoneurons
		−2.39	−2.37	Glia			1.07	Ventral horn (Jiang *et al.* 2005)
		−2.06	−1.99	MN+glia				
1635607_at	*CG31742*	−1.74	−1.81	MN	Proteasome (prosome, macropain) subunit, beta type, 5	human	1.2	Motoneurons
		−1.94	−1.68	Glia			1.42	Ventral horn (Jiang *et al.* 2005)
		−2.12	−1.78	MN+glia				
1623503_at	Slamdance	1.02*^a^*	−1.74	MN	Alanyl (membrane) aminopeptidase	human	0.61	Motoneurons
		−1.14*^a^*	−2.02	Glia			0.57	Ventral horn (Jiang *et al.* 2005)
		−1.04*^a^*	−2.26	MN+glia				

Mammalian orthologs of *Drosophila* genes that also have been identified as differentially regulated in other microarray experiments. MN, motoneurons.

a Not statistically significant at a Benjamini-Hochberg corrected *P* value of >0.05.

## Discussion

Here we examined the phenotypic effects of G85R and dSOD1 expression in motoneurons, glia, and simultaneously in motoneurons plus glia of flies. We have shown that cell-specific expression of dSOD1 and G85R can influence lifespan, sensitivity to hydrogen peroxide, and lipid peroxidation. Our microarray experiment has identified a number of candidate genes and enriched GO terms which may provide further insights into the cellular and molecular mechanisms by which mutant SOD1 affects neuronal and glial function in flies.

Cell-specific expression of SOD1 appears to confer a beneficial effect under certain circumstances but is harmful in other cases. Overexpression of wild-type dSOD1 has no effect on lifespan when expressed in motoneurons but decreases lifespan when over expressed in glia, indicating that abnormal expression of wild-type SOD1 may have toxic consequences in certain cells. While we find that G85R expression in motoneurons slightly decreases lifespan, motoneuronal expression of the G41S allele was previously reported to increase lifespan (Elia *et al.* 1999). G85R expressed in glia also decreases lifespan, but, when expressed simultaneously in motoneurons and glia, it increases the lifespan of flies. When motor function was measured in a negative geotaxis assay, glial expression of mutant SOD1 decreased climbing ability, whereas expression of mutant SOD1 in motoneurons and coexpression in motoneurons and glia enhanced climbing ability (Islam *et al.* 2012).

Flies with mutant SOD1 expression have also been shown to have a differential response to certain environmental toxins. We have shown that flies expressing G85R under motoneuronal and glial Gal4 drivers are more sensitive to hydrogen peroxide. However, our earlier study also showed that flies with cell-specific expression of G85R responded differently to BMAA and paraquat, suggesting toxin-specific interaction with mutant SOD1. Expression of the mutant human SOD1 in motoneurons alone or together with glial expression was shown to increase resistance to BMAA; when restricted to glia alone, it increased sensitivity to BMAA (Islam *et al.* 2012). Other investigators have shown that motoneuronal expression of G41S increased resistance to paraquat (Elia *et al.* 1999) or that G85R and A4V expressed in motoneurons, glia, or both had no significant effect on paraquat resistance (Islam *et al.* 2012). However, we do not know whether the difference to paraquat can be explained by different mutant SOD1 (G85R and A4V *vs.* G41S), or experimental conditions (20 mM paraquat in cornmeal fly food *vs.* 40 mM paraquat in 1% sucrose). Additionally, in a mouse study, the cre-lox system was used to excise mutant SOD1 expression from Schwann cells, which are glial cells of the peripheral nervous system. Removal of the mutant protein accelerated the disease, suggesting that its presence in Schwann cells slows disease progression (Lobsiger *et al.* 2009). These contradictory phenotypes may be an indication of hormesis, a term used to describe the phenomena of how exposure to a low dose to a toxic agent can confer a benefit, such as resistance to future stress, by activating repair mechanisms (Calabrese *et al.* 2011). Studies have shown that young flies exposed to low doses of H_2_O_2_ and other stresses, such as heat shock and hypergravity, have an increased ability to tolerate stress at an older age and live longer than flies not subjected to stress at a young age (Hercus *et al.* 2003; Le Bourg 2007; Le Bourg and Minois 1997; Le Bourg *et al.* 2001). Additionally, increased levels of ROS have been demonstrated to increase lifespan of other organisms, such as *Caenorhabditis elegans* (Yang and Hekimi 2010). By restricting mutant SOD1 expression to certain cells, whatever stress occurring there may be low enough to be hormetic when expressed in some cell types but is harmful in others. These opposing phenotypes are intriguing and further investigation may help us understand why motoneurons are the affected tissue in ALS and what role glia play in this disease.

Our microarray analysis examining changes in gene expression as a consequence of G85R expression is novel because the Gal4-UAS binary method affords us the ability to express G85R in specific cells. Although motoneurons or glia could be enriched to perform the microarray analysis, we believe the data obtained from RNA isolated from whole flies is valuable for obtaining a global view of transcriptional changes when mutant SOD1 is expressed in a cell -specific manner. It is known that degeneration of motoneurons is a key feature of ALS but changes at neuromuscular junctions have been reported to precede motoneuron loss (Fischer *et al.* 2004). However, it is important to stress that we only know the cell type in which we over expressed G85R, but we do not know which cell type is responsible for the transcriptional changes of the genes. Additionally, due to the cost of microarrays, we only used dSOD1 as a control. The *Drosophila* SOD1 was selected as a control because mutant human SOD1 has been shown to retain some activity in flies (Elia *et al.* 1999; Mockett *et al.* 2003). Additionally, wild-type human SOD1 was not used as a control because it has been shown to behave like a mutant SOD1 in flies (Islam *et al.*2012; Watson *et al.* 2008). It is, therefore, expected that some changes in gene expression may be masked by dSOD1 overexpression in comparison with G85R.

Analysis of our microarray data reveals the differential regulation of many genes and enriched GO terms when G85R is expressed in a cell-specific manner. Some of these genes and GO terms are unique to G85R expression in certain cell types and others are common among several or all cell type expression patterns of G85R. Genes uniquely regulated by motoneurons and glia are of particular interest because it suggests that coexpressing mutant SOD1 in both cells somehow cancels out the effect of expression in a single cell type.

Oxidation-reduction is a GO term commonly enriched in the down-regulated set of genes for most flies. Additionally, the pentose-phosphate pathway, NADP metabolism, and glutathione metabolism have been identified among the enriched the GO terms in down-regulated gene sets. Glutathione is an important antioxidant whose activity relies on NAPDH, a product of the pentose-phosphate pathway. Glutathione normally exists in its reduced state (GSH), but when under conditions of oxidative stress, it is oxidized to glutathione disulfide (GSSG). NADPH regenerates GSH from GSSG. Reduced expression of genes within these pathways may result in an impaired response to ROS. Consistent with this hypothesis, all older flies expressing G85R have an increased sensitivity to hydrogen peroxide and those with G85R expression in motoneurons and glia have evidence of increased oxidative stress as determined by lipid peroxidation levels. Taken together, these results suggest that oxidative stress may be an important consequence of expressing mutant SOD1 in flies.

Symptoms of ALS do not manifest until later in life and may be the result of cumulative damage that occurs over many years. For that reason, we are also interested in identifying temporal changes in gene expression. The results of the meta-analysis largely reflected those results obtained by individually comparing G85R to dSOD1 at each respective time point. Interestingly, most significant changes in old flies generally occurred in the down-regulated gene lists.

We have also identified several fly genes that are orthologs of mammalian genes reported as differentially regulated in SOD1 transgenic mouse and in human ALS patients. Because changes in gene expression in a subset of cells may not represent global changes in expression, it is not unexpected that some of the orthologous genes were oppositely regulated as we used whole flies compared to cultured cells and tissues harvested by laser capture microdissection. Importantly, these differentially expressed orthologs exemplify how flies are very useful for identifying potential biomarkers and global targets for therapeutic treatments.

Microarray analysis of cell-specific expression of mutant SOD1 in the motoneurons and glia of flies has provided us with a number of candidate genes for future investigation. These genes encompassed a variety of functions, including several of the molecular mechanisms believed to contribute to the pathology of ALS. It is noteworthy that even though mutant SOD1 was expressed in a subset of neuronal cells, we were able to detect relatively large changes in expression of genes that function within the nervous system. This set of microarrays has generated a vast amount of data and further work is needed to identify those genes that are most important. By making our results publicly available, we hope to assist other researchers in better understanding how mutant SOD1 contributes to ALS.

## Supplementary Material

Supporting Information
