# Supplementary material for: Transcriptome Profiling Following Neuronal and Glial Expression of ALS-Linked SOD1 in Drosophila
Source: G3 (Bethesda). 2013 Apr 1;3(4):695–708. doi: 10.1534/g3.113.005850 (PMC3618356; doi:10.1534/g3.113.005850)
Supplement: Supporting Information [file supp_3_4_695__index.html]

Transcriptome Profiling Following Neuronal and Glial Expression of ALS-Linked SOD1 in Drosophila — Supporting Information 

# Transcriptome Profiling Following Neuronal and Glial Expression of ALS-Linked SOD1 in *Drosophila*

## Supporting Information for Kumimoto, Fore, and Zhang, 2013

**Files in this Data Supplement:**

- Supporting Information - Files S1-S4 and Tables S1-S2 (PDF, 91 KB)
- Table S1 - Primers for real time RT-PCR (PDF, 57 KB)
- Table S2 - Mating scheme for cell-specific expression of SOD1 (PDF, 53 KB)
- File S1 - Genes with at least a 2-fold change in expression in G85R flies relative to their respective dSOD1 controls (.xlsx, 54 KB)
- File S2 - Comparison of quantitative RT-PCR results vs. microarray results. Changes in expression were determined by comparing G85R expressing flies to their dSOD1 controls. (.xlsx, 16 KB)
- File S3 - Enriched gene ontology terms in flies expressing G85R relative to their respective dSOD1 controls (.xlsx, 47 KB)
- File S4 - Enriched gene ontology terms in old G85R expressing flies from the meta-analysis (.xlsx, 16 KB)
