# Supplementary material for: Transcriptome Profiling Following Neuronal and Glial Expression of ALS-Linked SOD1 in Drosophila
Source: G3 (Bethesda). 2013 Apr 1;3(4):695–708. doi: 10.1534/g3.113.005850 (PMC3618356; doi:10.1534/g3.113.005850)
Supplement: Supporting Information [file supp_g3.113.005850_005850SI.pdf]

G3 · Genes | Genomes | Genetics

Transcriptome Profiling following Neuronal and Glial Expression of ALS-linked SOD1 in *Drosophila*

Emily L. Kumimoto, Taylor R. Fore, and Bing Zhang*[1]
Department of Biology
University of Oklahoma
Norman, OK 73019

*To whom correspondence should be sent (zhangbing@missouri.edu)
[1]Current address: Division of Biological Sciences, University of Missouri, Columbia, MO 65211-7400

DOI: 10.1534/g3.113.005850

Files S1-S4

Available for download at http://www.g3journal.org/lookup/suppl/doi:10.1534/g3.113.005850/-/DC1

File S1 Genes with at least a 2-fold change in expression in G85R flies relative to their respective dSOD1 controls.

File S2 Comparison of quantitative RT-PCR results vs. microarray results. Changes in expression were determined by comparing G85R expressing flies to their dSOD1 controls.

File S3 Enriched gene ontology terms in flies expressing G85R relative to their respective dSOD1 controls.

File S4 Enriched gene ontology terms in old G85R expressing flies from the meta-analysis.

Table S1   Primers for real time RT-PCR

GENE		SEQUENCE
CG13551	5'	CGCCTTAAGAACGACCAGAT
CG13551	3'	TCACTTGTGCAGGTTCTCG
CG31742	5'	CGTCGGGAAAGTTAATTGGA
CG31742	3'	AGCTCGATCCCAGTATGTGC
CG33296	5'	TGTGGTCTTGCAATCTGCTC
CG33296	3'	TTGGCCATAGATCCACAACA
heat shock protein 22	5'	TGGCTATAGCTCCAGGCACT
heat shock protein 22	3'	CGCTCCTTGAGTGTCTCCTG
longitudinals lacking	5'	ATGCCGGAGTTGTGGTAAAG
longitudinals lacking	3'	GTCATCGTATTCGGCTTTGG
Niemann-Pick type C-1b	5'	ACTCACTGTCCGTCCAGCTT
Niemann-Pick type C-1b	3'	CGGTGGTGACGTTGTACTTG
Niemann-Pick type C-2e	5'	ATCTCCTGTACGGTGCCATC
Niemann-Pick type C-2e	3'	GCATCGTTCAACGTGACAGT
cAMP-dependent protein kinase 1	5'	GGATTGCGATCTTCCAAAAG
cAMP-dependent protein kinase 1	3'	AGCAAACTCCTTGGCACACT
pointed	5'	ACGCCCTATGATGCTCAATC
pointed	3'	TATCCAGACCCAAGGTGCTC
Prosap	5'	CCCAAGACTATTCCCGATCA
Prosap	3'	GCTGTTGCACAAGTTGCTTC
Protein tyrosine phosphatase 99A	5'	ACTATGTGAGCCGCGACTTT
Protein tyrosine phosphatase 99A	3'	AGATGCTGTTGGGATTGGAC
rhomboid	5'	GTCCCCAGGTGTCGTACATT
rhomboid	3'	AACGCTAGCCACCAGATGAG
Ribosomal protein L32	5'	CGGATCGATATGCTAAGCTGT
Ribosomal protein L32	3'	GCGCTTGTTCGATCCGTA

Sema-1a	5'	CTGCTGGTCGGCTTCTTTAC
Sema-1a	3'	ACAGGACGAGGGGAAGCTAT
SCAP	5'	ACGAGAGGATTTGCGTATGG
SCAP	3'	CGCACATCCCACACAATAAG
sulfateless	5'	ACGGCGATGTTATAGCCAAC
sulfateless	3'	GATAGTAGGCCAGCCAGTGC
slamdance	5'	TCAAGCAGATCATGGACTCG
slamdance	3'	TCTGATCCGCAGTGTTCTTG
tumbleweed	5'	TTGGCCTCTATCGATTGTCC
tumbleweed	3'	GATATCCGTGTTGCCCAAAT
wrapper	5'	CTGAATCGGAGCTTCAGGAC
wrapper	3'	GAGCCCGAGTTGAACATCAT

Table S2 Mating scheme for cell-specific expression of SOD1

CELL TYPE	STATUS	MATING SCHEME	REPLICATES	AGES
Motoneuron	Control	D42-Gal4 X UAS-dSOD1^{wt}	3	5d, 45d
Motoneuron	Experimental	D42-Gal4 X UAS-hSOD1^{G85R}	3	5d, 45d
Glia	Control	M1B-Gal4 X UAS-dSOD1^{wt}	3	5d, 45d
Glia	Experimental	M1B-Gal4 X UAS-hSOD1^{G85R}	3	5d, 45d
Motoneuron + Glia	Control	D42-Gal4, M1B-Gal4 X UAS-dSOD1^{wt}	3	5d, 45d
Motoneuron + Glia	Experimental	D42-Gal4, M1B-Gal4 X UAS-hSOD1^{G85R}	3	5d, 45d

Mating scheme for cell-specific expression of SOD1. The UAS-Gal4 system in was used to drive cell specific expression of SOD1. To drive expression of SOD1 in motoneurons, flies containing the D42-Gal4 driver were crossed to flies containing UAS-dSOD1^{wt} and UAS-hSOD1^{G85R}. To drive expression of SOD1 in glia, flies containing the M1B-Gal4 driver were crossed to flies containing UAS-dSOD1^{wt} and UAS-hSOD1^{G85R}. To drive expression of SOD1 in motoneurons,and glia, flies containing both the D42-Gal4, M1B-Gal4 drivers were crossed to flies containing UAS-dSOD1^{wt} and UAS-hSOD1^{G85R}. Three biological replicates consisting of 40 adult male flies were harvested at 5 days and 45 days post eclosion.
