# Supplementary material for: Transcriptome Profiling Following Neuronal and Glial Expression of ALS-Linked SOD1 in Drosophila
Source: G3 (Bethesda). 2013 Apr 1;3(4):695–708. doi: 10.1534/g3.113.005850 (PMC3618356; doi:10.1534/g3.113.005850)
Supplement: Supporting Information [file supp_g3.113.005850_TableS1.pdf]

Table S1 Primers for real time RT-PCR

GENE		SEQUENCE
CG13551	5'	CGCCTTAAGAACGACCAGAT
CG13551	3'	TCACTTGTGCAGGTTCTCG
CG31742	5'	CGTCGGGAAAGTTAATTGGA
CG31742	3'	AGCTCGATCCCAGTATGTGC
CG33296	5'	TGTGGTCTTGCAATCTGCTC
CG33296	3'	TTGGCCATAGATCCACAACA
heat shock protein 22	5'	TGGCTATAGCTCCAGGCACT
heat shock protein 22	3'	CGCTCCTTGAGTGTCTCCTG
longitudinals lacking	5'	ATGCCGGAGTTGTGGTAAAG
longitudinals lacking	3'	GTCATCGTATTCGGCTTTGG
Niemann-Pick type C-1b	5'	ACTCACTGTCCGTCCAGCTT
Niemann-Pick type C-1b	3'	CGGTGGTGACGTTGTACTTG
Niemann-Pick type C-2e	5'	ATCTCCTGTACGGTGCCATC
Niemann-Pick type C-2e	3'	GCATCGTTCAACGTGACAGT
cAMP-dependent protein kinase 1	5'	GGATTGCGATCTTCCAAAAG
cAMP-dependent protein kinase 1	3'	AGCAAACTCCTTGGCACACT
pointed	5'	ACGCCCTATGATGCTCAATC
pointed	3'	TATCCAGACCCAAGGTGCTC
Prosap	5'	CCCAAGACTATTCCCGATCA
Prosap	3'	GCTGTTGCACAAGTTGCTTC
Protein tyrosine phosphatase 99A	5'	ACTATGTGAGCCGCGACTTT
Protein tyrosine phosphatase 99A	3'	AGATGCTGTTGGGATTGGAC
rhomboid	5'	GTCCCCAGGTGTCGTACATT
rhomboid	3'	AACGCTAGCCACCAGATGAG
Ribosomal protein L32	5'	CGGATCGATATGCTAAGCTGT
Ribosomal protein L32	3'	GCGCTTGTTCGATCCGTA

Sema-1a	5'	CTGCTGGTCGGCTTCTTTAC
Sema-1a	3'	ACAGGACGAGGGGAAGCTAT
SCAP	5'	ACGAGAGGATTTGCGTATGG
SCAP	3'	CGCACATCCCACACAATAAG
sulfateless	5'	ACGGCGATGTTATAGCCAAC
sulfateless	3'	GATAGTAGGCCAGCCAGTGC
slamdance	5'	TCAAGCAGATCATGGACTCG
slamdance	3'	TCTGATCCGCAGTGTTCTTG
tumbleweed	5'	TTGGCCTCTATCGATTGTCC
tumbleweed	3'	GATATCCGTGTTGCCCAAAT
wrapper	5'	CTGAATCGGAGCTTCAGGAC
wrapper	3'	GAGCCCGAGTTGAACATCAT
