# Supplementary material for: Transcriptome Profiling Following Neuronal and Glial Expression of ALS-Linked SOD1 in Drosophila
Source: G3 (Bethesda). 2013 Apr 1;3(4):695–708. doi: 10.1534/g3.113.005850 (PMC3618356; doi:10.1534/g3.113.005850)
Supplement: Supporting Information [file supp_g3.113.005850_TableS2.pdf]

Table S2 Mating scheme for cell-specific expression of SOD1

CELL TYPE	STATUS	MATING SCHEME	REPLICATES	AGES
Motoneuron	Control	D42-Gal4 X UAS-dSOD1^{wt}	3	5d, 45d
Motoneuron	Experimental	D42-Gal4 X UAS-hSOD1^{G85R}	3	5d, 45d
Glia	Control	M1B-Gal4 X UAS-dSOD1^{wt}	3	5d, 45d
Glia	Experimental	M1B-Gal4 X UAS-hSOD1^{G85R}	3	5d, 45d
Motoneuron + Glia	Control	D42-Gal4, M1B-Gal4 X UAS-dSOD1^{wt}	3	5d, 45d
Motoneuron + Glia	Experimental	D42-Gal4, M1B-Gal4 X UAS-hSOD1^{G85R}	3	5d, 45d

Mating scheme for cell-specific expression of SOD1. The UAS-Gal4 system in was used to drive cell specific expression of SOD1. To drive expression of SOD1 in motoneurons, flies containing the D42-Gal4 driver were crossed to flies containing UAS-dSOD1^{wt} and UAS-hSOD1^{G85R}. To drive expression of SOD1 in glia, flies containing the M1B-Gal4 driver were crossed to flies containing UAS-dSOD1^{wt} and UAS-hSOD1^{G85R}. To drive expression of SOD1 in motoneurons,and glia, flies containing both the D42-Gal4, M1B-Gal4 drivers were crossed to flies containing UAS-dSOD1^{wt} and UAS-hSOD1^{G85R}. Three biological replicates consisting of 40 adult male flies were harvested at 5 days and 45 days post eclosion.
